# Evolutionary Context of Non–Sorbitol-Fermenting Shiga Toxin–Producing *Escherichia coli* O55:H7

**DOI:** 10.3201/eid2312.170628

**Published:** 2017-12

**Authors:** Kyle Schutz, Lauren A. Cowley, Sharif Shaaban, Anne Carroll, Eleanor McNamara, David L. Gally, Gauri Godbole, Claire Jenkins, Timothy J. Dallman

**Affiliations:** Public Health England Colindale, London, UK (K. Schutz, L.A. Cowley, G. Godbole, C. Jenkins, T.J. Dallman);; The University of Edinburgh, Midlothian, UK (S. Shaaban, D.L. Gally);; Cherry Orchard Hospital, Dublin, Ireland (A. Carroll, E. McNamara)

**Keywords:** Shiga toxin–producing *Escherichia coli* serotype, STEC O55:H7, outbreak, whole-genome sequencing, virulence, evolution, bacteria, England, Ireland, zoonoses

## Abstract

In July 2014, an outbreak of Shiga toxin–producing *Escherichia coli* (STEC) O55:H7 in England involved 31 patients, 13 (42%) of whom had hemolytic uremic syndrome. Isolates were sequenced, and the sequences were compared with publicly available sequences of *E. coli* O55:H7 and O157:H7. A core-genome phylogeny of the evolutionary history of the STEC O55:H7 outbreak strain revealed that the most parsimonious model was a progenitor enteropathogenic O55:H7 sorbitol-fermenting strain, lysogenized by a Shiga toxin (Stx) 2a–encoding phage, followed by loss of the ability to ferment sorbitol because of a non-sense mutation in *srlA*. The parallel, convergent evolutionary histories of STEC O157:H7 and STEC O55:H7 may indicate a common driver in the evolutionary process. Because emergence of STEC O157:H7 as a clinically significant pathogen was associated with acquisition of the Stx2a-encoding phage, the emergence of STEC O55:H7 harboring the *stx2a* gene is of public health concern.

The first outbreak of Shiga–toxin producing *Escherichia coli* (STEC) O55:H7 in the United Kingdom occurred in the county of Dorset, England, in July 2014 (*1*). Ultimately, 31 cases were linked to the outbreak, and 13 (42%) of those patients had hemolytic uremic syndrome (HUS). Of the 13 with HUS, 8 (66%) had neurologic complications and 11 (90%) required prolonged treatment for kidney replacement. After enhanced epidemiologic surveillance and analysis of the patients’ food, exposure, and travel histories, the only epidemiologic link identified was living in or having close links to Dorset County. Extensive microbiological investigations included testing of the environment, nondomestic animals, and household pets. Although no causal link was established, whole-genome sequencing and epidemiologic analyses were indicative of a local endemic zoonotic source (*1*).

Previous studies postulated that the common STEC O157:H7 clone evolved from enteropathogenic *E. coli* (EPEC) serotype O55:H7 (*2*,*3*). Evolutionary models predict the stepwise acquisition of a Shiga toxin (Stx)–encoding bacteriophage in the EPEC O55:H7 progenitor strain, followed by the substitution of the *rfb* locus encoding the somatic O55 antigen with that encoding the O157 antigen, the acquisition of the pO157 plasmid, loss of the ability to ferment sorbitol, and loss of the ability to produce β-glucuronidase (*3*–*6*). Analyses from more recent studies have indicated that the Stx-encoding phage is an unstable evolutionary marker, with frequent acquisition and loss occurring in STEC O55:H7 and all 3 lineages of STEC O157 throughout their evolutionary history (*7*,*8*).

STEC O157:H7 has multiple genetic and phenotypic features that contribute to its pathogenicity or are used for detection and identification. The primary virulence factor defining the STEC group is production of Stx1, Stx2, or both. The genes encoding the toxins, *stx1* and *stx2*, are harbored on lambdoid prophage and are the targets of commercial and in-house diagnostic PCR assays (*9*). Both toxins can be divided into several subtypes, Stx1a–1d and Stx2a–2g (*10*). The locus of enterocyte effacement (LEE) is a 35-kb pathogenicity island encoding a type III secretion system (T3SS) responsible for the attaching and effacing phenotype that facilitates successful colonization of the human gut (*11*). The inability to ferment sorbitol or to produce β-glucuronidase differentiates STEC O157 from ≈90% of other gastrointestinal bacteria (*5*,*12*). These characteristics, along with resistance to tellurite, facilitate the detection and identification of STEC O157:H7 on selective media. The pO157 plasmid encodes multiple putative virulence factors, including enterohemolysin (*ehxA*) and an adhesin (*toxB*) (*13*).

The STEC O55:H7 Dorset outbreak strain shared certain characteristics with the STEC O157:H7 clone. Initial PCRs detected the presence of *stx2* and the intimin gene *eae*, a marker for *E. coli* attaching and effacing phenotype; non–sorbitol-fermenting colonies of STEC O55 were identified after culture on sorbitol MacConkey agar (*1*,*9*). However, unlike the STEC O157 clone, the STEC O55 Dorset outbreak strain exhibited β-glucuronidase activity and was sensitive to tellurite. Laboratory records held at the Gastrointestinal Bacterial Reference Unit of Public Health England showed that this highly pathogenic strain had not previously been isolated from humans or animals in the United Kingdom. Our goal with this study was to identify the genetic determinants responsible for the phenotypic characteristics of the STEC O55:H7 Dorset outbreak strain and to explore the strain’s evolutionary history.

## Materials and Methods

### Bacterial Strains

We studied 26 isolates of STEC O55:H7 from the outbreak, 10 isolates of STEC O55:H7 from Ireland, and 79 isolates selected to represent of the broad phylogeny of STEC O157:H7 (Technical Appendix Table). From public databases, we retrieved 10 genome sequences for *E. coli* O55:H7 and 2 for STEC O157:H7 (*6*,*7*,*14*,*15*) (Table 1).

**Table 1:** *Escherichia coli* O55:H7 genome sequences retrieved from publicly available databases*

Name	Accession no.	Serotype	STX	SOR	GUD	Reference
USDA 5905	SRS702210	O55	–	+	+	(*7*)
3256–97–1	AEUA01000000	O55	–	+	+	(*7*)
RM12579–1	CP003109	O55	–	+	+	(*7*)
CB9615	NC_013941	O55	–	+	+	(*10*)
ZH-1141	Pending	O55:H7	–	+	+	(*14*)
2013C-4465	GCA_001644745.1	O55	Stx1a	+	+	(*15*)
Sakai		O157:H7	Stx1a and 2a	–	–	(*16*)
155	CP018237	O157:H7	Stx2a	–	–	(*17*)
TL-000142	ERR180875	O55	–	+	+	This study
SRR3578942	SRR3578942	O55:H7	Stx2d	+	+	This study
TL-000132	ERR197199	O55	–	+	+	This study
3041–1_85	ERR197201	O55	–	+	+	This study
100446	ERR178176	O55:H7	–	+	+	This study

*GUD, β-glucuronidase; SOR, sorbitol; Stx, Shiga toxin; –, negative; +, positive.

### Whole-Genome Sequencing, Assembly, and Alignment

We sequenced all isolates by using an Illumina paired-end (100-bp) protocol (https://www.illumina.com) and assembled them by using SPAdes Genome Assembler version 3.1.1 (*18*). The assemblies were annotated by using Prokka version 1.0.1 (*19*). We used the MinION (https://nanoporetech.com/products/minion) nanopore platform to sequence an isolate from the outbreak, designated 122262. A hybrid Illumina/MinION de novo assembly of 122262 constructed by using SPAdes yielded 15 contigs with the largest contig spanning the first 2.4 mbp. We aligned published reference genomes against the outbreak reference strain 122262 by using Mauve (*20*).

### Genome, Plasmid, and Bacteriophage Comparisons

We retrieved from GenBank published nucleotide sequences of key virulence genes associated with toxicity, host-cell adhesion, and metabolic activity and concatenated in FASTA (http://www.ebi.ac.uk/Tools/sss/fasta/) file format. To determine the presence and absence of the gene panel, we performed a blastn (*21*) comparison against the extracted coding sequences of 122262. Significant hits were defined as those with a nucleotide identity of >90% over at least 90% of the query sequence. Truncated sequences were defined as matches with <90% coverage. We uploaded assembled data from the strains in FASTA file format to the PHAge Search Tool (PHAST) web server for prophage identification (*22*). Prophage region detection, prophage annotation, and circular genomic views from PHAST results were used along blast ring image generator (BRIG) plots (*23*) to isolate the prophage regions of 122262 and nucleotide homologies to the prophages in the Sakai reference genome (*16*). BRIG was used to visually compare the similarities between the Sakai and outbreak strain prophages. We compared prophage regions of 122262 with those extracted and analyzed by Shaaban et al. (*17*) by using the pipeline and strains presented in their study.

### Phylogenetic Analyses

Short reads were quality trimmed (*24*) and mapped to the STEC O157:H7 Sakai reference genome (GenBank accession no. BA000007) by using Burrows-Wheeler aligner–maximal exact matching (*25*). We sorted and indexed the sequence alignment map output from the Burrows-Wheeler aligner to produce a binary alignment map by using SAMtools (*25*). GATK2 (*26*) was used to create a variant call format file from each of the B binary alignment maps, which were further parsed to extract only single-nucleotide polymorphism (SNP) positions that were of high quality (mapping quality >30, coverage of reads that passed quality metrics >10, variant ratio >0.9). We used pseudosequences of polymorphic positions to create maximum-likelihood trees by using RAxML (*27*). FASTQ (https://www.ncbi.nlm.nih.gov/pmc/articles/PMC2847217/) sequences were deposited in the National Center for Biotechnology Information Short Read Archive under the BioProject PRJNA248042.

## Results

### General Genomic Features

STEC O55:H7 strain 122262 had a 5,364,131-bp chromosome and a 67,247-bp single plasmid of replicon type FIB-15. Use of blastn to compare the extracted plasmid sequence from 122262 with publicly available plasmid sequences belonging to CB9615, 2013C-4465, and Sakai indicated that the plasmid of 122262 was 99% identical to pO55 CB9615 over its complete length. Unlike pO157 in STEC O157:H7, the O55:H7 plasmids did not encode toxin B (*toxB*) or the enterohemolysin operon (*ehxABCD*). The *E. coli* O55:H7 strains 122262, CB9615, and 2013C-4465 did, however, encode a remote *toxB* homologue *efa1*/*lifA* on the chromosome that has 29% nt identity (97% coverage) with pO157 *toxB.* The LEE was inserted into the chromosome of strain 122262 at *tRNA-selC*, the most common insertion site in a range of pathogenic *E. coli* chromosomal backgrounds (*28*). Antimicrobial drug resistance determinants included *aadA-1b* encoding resistance to streptomycin and *dfrA-1* encoding resistance to trimethoprim.

### Prophage Composition of 122262

**Figure 1:**
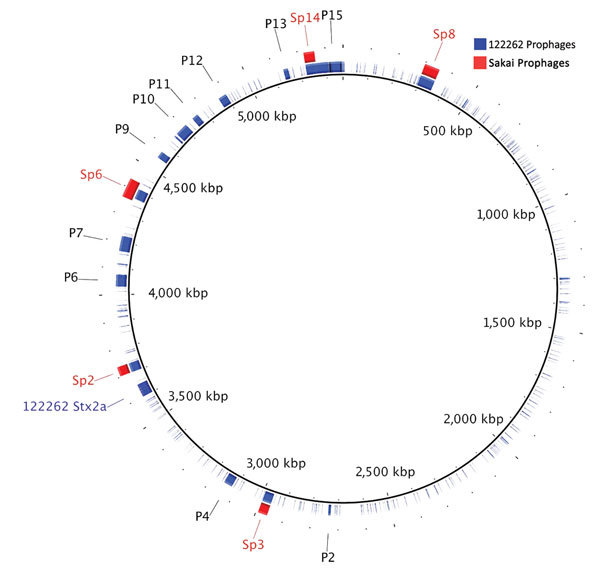
BLAST ring image generator (BRIG) plot generated from BLAST+ (*21*) comparisons of Shiga toxin–producing *Escherichia coli* (STEC) O55:H7 122262 prophages and homologous STEC O157:H7 Sakai prophages. STEC O55:H7 122262 chromosome is set as the reference genome, and the 122262 prophages (P1–P15) comprise the first ring. The homologous STEC O157:H7 Sakai prophages (Sp2, Sp3, Sp6, Sp8, and Sp14) identified in the BLAST analysis were added to the image according to their known locations (Table 2). Putative prophage sequence data were retrieved content from PHAge Search Tool (*22*) and plotted in BRIG.

PHAST identified 15 prophage interruptions in 122262, of which 5 were homologous in nucleotide identity to Sp2, Sp3, Sp6, Sp8, and Sp14 found in Sakai (*16*) (Table 2; Figure 1). Unique genetic content and position was found for 9 putative prophages (Figure 1). In addition, a Stx2a-encoding phage was identified at the Stx-associated bacteriophage insertion site *yecE* in strain 122262. In Sakai, the Stx1a (Sp15) and Stx2a (Sp5) encoding phages are inserted at *wrbA* and *yehV*, respectively. However, *yecE* is a known Stx-associated bacteriophage insertion site in strains of STEC O157:H7 encoding *stx2a* belonging to lineage Ic (*29*).

**Table 2:** Location of prophages in Shiga toxin–producing *Escherichia coli* O55:H7 isolate 122262 from outbreak in Dorset County, England, July 2014, and related Sakai reference prophage*

Prophage in 122262	Location	Related Sakai phage	Identity, %
P1	298714–355267	Sp8	96
P2	2728769–2738381	NP	NA
P3	2958215–2992979	Sp3	98
P4	3119806–3151485	NP	NA
P5	3702030–3736837	Sp5	99
P6	4031314–4075190	NP	NA
P7	4166735–4223146	NP	NA
P8	4361295–4432383	Sp6	97
P9	4549353–4575262	NP	NA
P10	4662955–4712352	NP	NA
P11	4744636–4768829	NP	NA
P12	4868835–4901248	NP	NA
P13	5136256–5154117	NP	NA
P14	5221278–5261127	Sp14	98
P15	5287889–5361495	NP	NA
Stx-encoding phage	3607500–3655000	NP	NA

*NA, not applicable; NP, not present; Stx, Shiga toxin.

**Figure 2:**
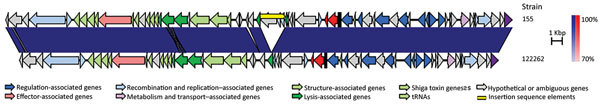
The sequence of the Stx2a-encoding phage from the July 2014 Dorset County, England, outbreak strain of Shiga toxin–producing *Escherichia* coli O55:H7, designated 122262, showed >98% nt identity with an outlier Stx2a-encoding phage designated 155, found in a subset of isolates of Shiga toxin–producing *Escherichia coli* O157 geographically associated with the island of Ireland. The main difference between the 2 prophages was an insertion sequence element.

Long-read sequencing of 122262 facilitated comparison of the sequence of the Stx2a-encoding phage with other publicly available sequences of Stx2a-encoding phage. Shaaban et al. (*17*) compared prophage sequences for 14 strains of STEC O157:H7, including 8 Stx2a-encoding phages. Of the 8 Stx2a phages described in that study, 7 were closely related despite being found in globally distributed strains from different lineages. The sequence of the Stx2a-encoding phage from the outbreak strain, 122262, showed most similarity (>98% nt identity and >94% sequence coverage over the complete phage) with an outlier Stx2a-encoding phage designated 155, found in a subset of isolates of STEC O157 phage type 32 in lineage 1c, geographically associated with the island of Ireland (*17*,*29*) (Figure 2). The main difference between the 2 prophages was an insertion sequence element, a common source of prophage variation (Figure 2).

### Sorbitol-Negative Phenotype of 122262

Like the common STEC O157:H7 clone, the STEC O55:H7 outbreak strain described in this study was characterized by its inability to ferment sorbitol. *srlA* and *srlE* encode components of a glucitol/sorbitol-specific phosphotransferase system. In STEC O157:H7, the sorbitol-negative phenotype was thought to have resulted from frameshifts in *srlA* and *srlE*, as observed in Sakai and EDL933 (*5*). SNP analysis of STEC O55:H7 122262 in our study revealed a non-sense mutation in *srlA* causing truncation of the last 29 aa, which was likely to reduce expression or produce a nonfunctional product. The sorbitol-negative phenotype, although a characteristic of STEC O157:H7, is rare in *E. coli* O55:H7 and has been described for only 1 other strain (RM12506, also referred to as BB2 and C523-03; genome not publicly available) (*7*,*30*).

### β-Glucuronidase and Tellurite Phenotypes of 122262

β-glucuronidase is an inducible enzyme encoded by *uidA* and produced by ≈90% of pathogenic and nonpathogenic *E. coli*. The common STEC O157:H7 clone is a rare exception. The *uidA* loss of function mechanism in STEC O157:H7 was elucidated by Monday et al. (*31*) and included 2 frameshift mutations. The STEC O55:H7 outbreak strain 122262 had a β-glucuronidase–positive phenotype, and analysis of the genome by using MAUVE (*20*) did not identify any disruptive mutations in *uidA*. No β-glucuronidase–negative strains of *E. coli* O55:H7 have been described. Furthermore, the STEC O55 Dorset outbreak strain 122262 did not contain the *ter* cluster and was phenotypically sensitive to tellurite. As a consequence, it did not propagate when inoculated onto cefixime and tellurite sorbitol MacConkey agar and was not detected by routine culture methods used at the local hospital diagnostic microbiology laboratories in the United Kingdom (https://www.gov.uk/government/publications/smi-b-30-investigation-of-faecal-specimens-for-enteric-pathogens).

### Phylogenetic Analyses

**Figure 3:**
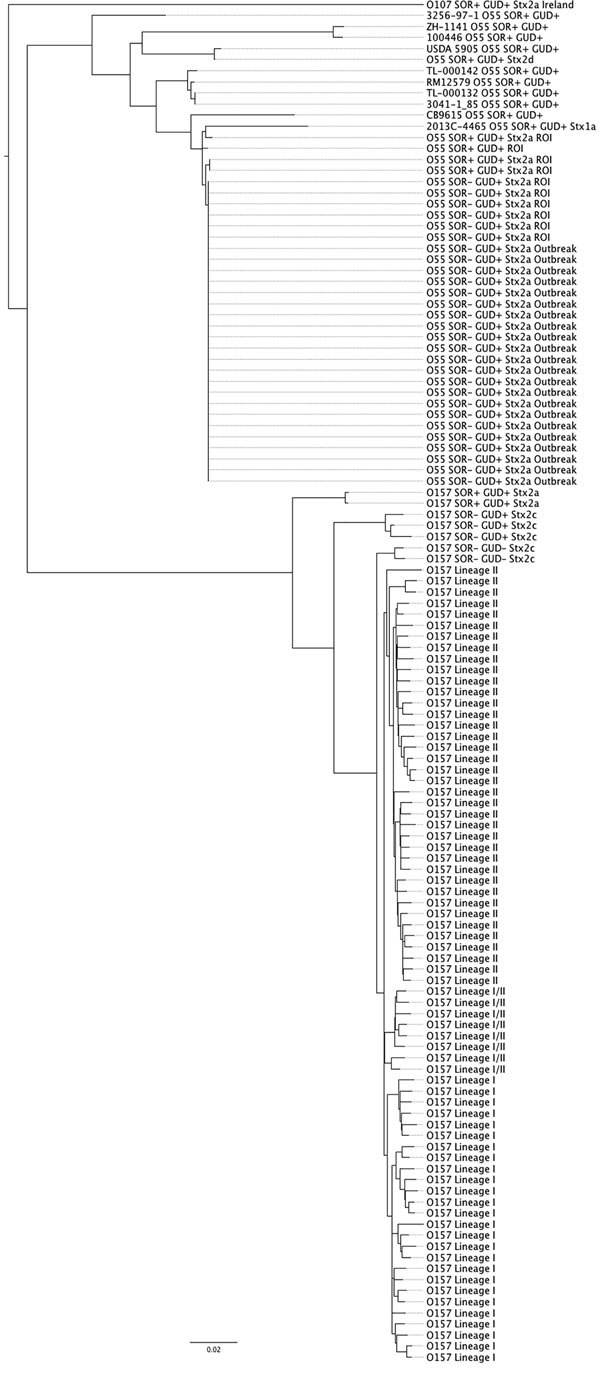
Core genome phylogeny illustrating the evolutionary history of the of Shiga toxin–producing *Escherichia coli* (STEC) O55 strain from the July 2014 Dorset County, England, outbreak in the context of STEC O157:H7 lineages I, II, and I/II. Scale bar indicates nucleotide substitutions per site.

To investigate the evolutionary history of the STEC O55 Dorset outbreak strain, we constructed a core genome phylogeny (Figure 3). The analysis divided the sequences of the isolates in this study according to serotype; all isolates of *E. coli* O55:H7 clustered together on a separate branch of the tree, and all isolates of STEC O157:H7 clustered together on the branch below, regardless of sorbitol/β-glucuronidase phenotype or the presence of *stx* (Figure 3). The phylogenetic analysis of *E. coli* O55:H7 indicated that incorporation of the Stx-encoding prophage has occurred on multiple occasions within the EPEC O55:H7 background, with independent acquisition of *stx1* (*15*), *stx2d*, and *stx2a* into EPEC O55:H7. Likewise, multiple acquisition and loss events involving *stx1*, *stx2c, stx2a*, and less commonly *stx2d* have been described for STEC O157:H7 (*12*,*32*).

As noted by McFarland et al. (*1*), the outbreak strain was closely related to STEC O55:H7 *stx2a* isolates identified in Ireland during 2013–2014 (Figure 3). These 6 isolates from Ireland were <5 SNPs from the Dorset outbreak strain, indicating that the isolates from Ireland and Dorset County shared a common source (*8*). The outbreak strain had lost the ability to ferment sorbitol, which appears to be a recent adaption with all ancestral O55:H7, including those isolated in Ireland in 2012 retaining the ability to ferment sorbitol. A similar relationship exists between the sorbitol-positive and sorbitol-negative STEC O157:H7 phenotypes; the sorbitol-negative phenotype is a more recent adaption from the sorbitol-positive progenitor strain (Figure 3) (*3*,*5*).

The most parsimonious model of evolution of the STEC O55:H7 Dorset outbreak strain was a progenitor EPEC O55:H7 sorbitol-fermenting strain lysogenized by an Stx2a-encoding phage and subsequent loss of the ability to ferment sorbitol. This stepwise model of evolution seems to mirror that seen in the common STEC O157:H7 clone; the acquisition of the STEC pathotype preceded phenotypic modulation.

## Discussion

In the United Kingdom, STEC is regarded as a substantial threat to public health, and enhanced surveillance systems are in place (*32*). In England, HUS developed in ≈5% of symptomatic STEC O157:H7 patients (*33*), notably less than the 42% of patients in whom HUS developed during the STEC O55:H7 outbreak described in this study. The Dorset outbreak strain was closely related to the common STEC O157:H7 clone and shared several characteristics, most notably the presence of phage-encoded *stx2a*. Stx2a is associated with more severe symptoms, including the development of HUS, and it is probably the key virulence factor causing the high proportion of HUS cases in this outbreak (*10*). Of additional concern was the inability to detect the outbreak strain at the local hospital level by using the standard microbiology investigation method, cefixime and tellurite sorbitol MacConkey agar, because of this strain’s sensitivity to tellurite.

A previously published stepwise evolutionary model showed the acquisition of *stx2* by a strain of EPEC O55:H7, resulting in emergence of a strain of STEC O55:H7, which was β-glucuronidase positive and sorbitol positive, closely related but ancestral to STEC O157:H7, which was β-glucuronidase positive and sorbitol positive (*34*). The loss of the sorbitol-positive phenotype in STEC O157:H7 was followed by the loss of β-glucuronidase expression, resulting in the common STEC O157 sorbitol-negative β-glucuronidase–negative clone. The evolutionary history of the Dorset outbreak strain begins with the EPEC O55:H7 progenitor strain described previously (*6*) (Figure 3). Subsequent acquisition of an Stx2a-encoding phage was confirmed by detection of STEC O55:H7 β-glucuronidase–positive sorbitol-positive isolates in Ireland in 2012 (Figure 3). The loss of the sorbitol-positive phenotype mirrored the genetic events proposed to have occurred in the evolution of STEC O157, albeit by an alternative mechanism.

The parallel, convergent evolutionary history of STEC O157:H7 and STEC O55:H7 may indicate a common driver in the evolutionary process. Adaptation to a new niche may be accompanied by modification of gene expression because genes no longer required for, or incompatible with, the variation in lifestyle are selectively inactivated by point mutation, insertion, or deletion (*35*). Loss of the sorbitol-positive phenotype may coincide with the successful colonization of a new animal host or the ability to transmit more effectively between animal hosts without the need to survive in the environment for long periods (*28*,*36*).

The detection of the STEC O55:H7 sorbitol-negative strain in patients in Ireland before the outbreak in Dorset led to speculation that ruminants (most likely cattle or sheep) on the island of Ireland were the source of the outbreak strain (*1*). Transmission between Ireland and Dorset may have occurred via movement of persons, livestock, or a secondary vector such as migratory birds (*37*). The finding that the Stx2a-encoding phage has a high level of similarity to Stx2a-encoding phage found in a previously described sublineage of STEC O157 PT32 geographically linked to Ireland may provide further evidence of the origin of this strain (*17*,*29*). Phages from STEC O157 may be exchanged with other phages from serotypes of *E. coli* in the gut of the ruminant host or in the environment. Analysis and comparison of phage sequences to provide clues regarding the origin of a strain of STEC is a novel approach to outbreak investigation; additional studies are required to evaluate the utility of the approach. Further work will be hampered by the lack of available sequences of the Stx-encoding phage and the difficulties with assembling the sequences because of the inability of short-read sequencing to resolve the large number of repetitive and paralogous features characteristic of the prophage.

The STEC O55:H7 Dorset outbreak strain described in this study shared characteristics with the common STEC O157:H7 clone, specifically the acquisition of an Stx2a-encoding phage and the sorbitol-negative phenotype. Key differences between the 2 strains include the *rfb* gene cluster, plasmid content, β-glucuronidase phenotype, and the absence of the *ter* gene cluster in the STEC O55:H7 outbreak strain. Despite these differences, this study provides evidence of parallel, convergent evolution of STEC O157:H7 and STEC O55:H7, involving multiple acquisitions of Stx-encoding phages and loss of the ability to ferment sorbitol. Previous studies have shown a clear association with STEC harboring *stx2a* and progression to HUS (*10*). Acquisition of the Stx2a-encoding phage seems to explain the emergence of STEC O157:H7 as a clinically significant pathogen; in contrast to the acquisition of *stx2c*, evidence suggests that after Stx2a-encoding phage is integrated in a population, it tends to be maintained and may be associated with higher excretions levels in cattle (*29*,*36*). As such, the emergence of STEC O55:H7 harboring *stx2a* is of public health concern.

Technical Appendix Genomic analysis of 26 isolates of Shiga toxin–producing *Escherichia coli* (STEC) O55:H7 from the July 2014 outbreak in Dorset County, England; 10 isolates of STEC O55:H7 from Ireland; and 79 isolates representing the broad phylogeny of STEC O157:H7.
