# Supplementary material for: Evolutionary Context of Non–Sorbitol-Fermenting Shiga Toxin–Producing Escherichia coli O55:H7
Source: Emerg Infect Dis. 2017 Dec;23(12):1966–73. doi: 10.3201/eid2312.170628 (PMC5708253; doi:10.3201/eid2312.170628)
Supplement: Technical Appendix — Genomic analysis of 26 isolates of Shiga toxin–producing Escherichia coli (STEC) O55:H7 from the July 2014 outbreak in Dorset County, England; 10 isolates of STEC O55:H7 from Ireland; and 79 isolates representing the broad phylogeny of STEC O157:H7. [file 17-0628-Techapp-s1.pdf]

Article DOI: https://doi.org/10.3201/eid2312.170628

Evolutionary Context of Non–Sorbitol-Fermenting Shiga Toxin–Producing *Escherichia coli* O55:H7

Technical Appendix

Technical Appendix Table. Genomic analysis of 26 isolates of STEC O55:H7 from the July 2014 outbreak in Dorset County, England; 10 isolates of STEC O55:H7 from Ireland, and 79 isolates selected to represent of the broad phylogeny of STEC O157:H7*

ACCESSION	SEROGROUP	STX	SOR	GUD	ANNOTATION
ERR178176	O55	–	+	+	100446 SOR+ GUD+
GCA_001644745.1	O55	stx1a	+	+	2013C–4465 SOR+ GUD+ Stx1a
ERR180875	O55	–	+	+	TL–000142 SOR+ GUD+
NC_013941	O55	–	+	+	CB9615 SOR+ GUD+
ADMX01000000	O157	stx2a	–	–	Lineage I
AKLW01000000	O157	stx1/2a/2c	–	–	Lineage I
AKLY01000000	O157	stx2c	–	–	Lineage I
AKMN01000000	O157	stx2c	–	–	Lineage I
SRR1984083	O157	stx2c	–	–	Lineage I
SRR4180231	O157	stx2c	–	–	Lineage I
SRR5904129	O157	stx2a/2c	–	–	Lineage I
SRR2034057	O157	stx1a/2c	–	–	Lineage I
SRR5024451	O157	stx2c	–	–	Lineage I
SRR5904130	O157	stx2a	–	–	Lineage I
SRR2187202	O157	stx2a/2c	–	–	Lineage I
SRR2187203	O157	stx2a/2c	–	–	Lineage I
SRR1981319	O157	stx2c	–	–	Lineage I
SRR1981361	O157	stx2c	–	–	Lineage I
SRR1981368	O157	stx2c	–	–	Lineage I
SRR1981423	O157	stx2c	–	–	Lineage I
SRR4176979	O157	stx2c	–	–	Lineage I
SRR4180824	O157	stx2c	–	–	Lineage I
SRR4184846	O157	stx2a	–	–	Lineage I
SRR4786402	O157	stx2a/2c	–	–	Lineage I
SRR4788235	O157	stx2a	–	–	Lineage I
SRR4788323	O157	stx2a	–	–	Lineage I
SRR5005300	O157	stx2c	–	–	Lineage I
SRR5006372	O157	stx1a/2c	–	–	Lineage I
SRR5024199	O157	–	–	–	Lineage I
SRS932722	O157	stx1/2a/2c	–	–	Lineage I
Not available	O157	stx1a/2c	–	–	Lineage I/II
SRR5904105	O157	stx1a/2c	–	–	Lineage I/II
SRR1981384	O157	stx2a/2c	–	–	Lineage I/II
SRR2034081	O157	stx1a/2c	–	–	Lineage I/II
SRR4787234	O157	stx2c	–	–	Lineage I/II
SRR4787274	O157	stx1a/2c	–	–	Lineage I/II
SRR4788195	O157	stx2c	–	–	Lineage I/II
SRR4897273	O157	stx1a/2c	–	–	Lineage I/II
AKKY01000000	O157	stx1a/2c	–	–	Lineage II
AKLA01000000	O157	stx1a/2c	–	–	Lineage II
SRR5905734	O157	stx2c	–	–	Lineage II
SRR5905747	O157	stx2c	–	–	Lineage II
SRR5018342	O157	–	–	–	Lineage II
SRR5904104	O157	stx2c	–	–	Lineage II
SRR1980985	O157	stx2c	–	–	Lineage II
SRR1981377	O157	stx2c	–	–	Lineage II
SRR1981397	O157	stx2c	–	–	Lineage II
SRR1981716	O157	stx2c	–	–	Lineage II
SRR2034093	O157	stx2c	–	–	Lineage II
SRR4177018	O157	stx2c	–	–	Lineage II

ACCESSION	SEROGROUP	STX	SOR	GUD	ANNOTATION
SRR4181527	O157	stx2c	–	–	Lineage II
SRR4184741	O157	stx2c	–	–	Lineage II
SRR4192121	O157	stx2a/2c	–	–	Lineage II
SRR4195486	O157	stx2c	–	–	Lineage II
SRR4195749	O157	stx2c	–	–	Lineage II
SRR4195765	O157	stx2a/2c	–	–	Lineage II
SRR4195765	O157	stx2a/2c	–	–	Lineage II
SRR4787238	O157	stx2a/2c	–	–	Lineage II
SRR4787292	O157	stx2c	–	–	Lineage II
SRR4787855	O157	stx2c	–	–	Lineage II
SRR4787900	O157	stx2c	–	–	Lineage II
SRR4787906	O157	stx2c	–	–	Lineage II
SRR4788210	O157	stx1a/2c	–	–	Lineage II
SRR4788290	O157	stx2c	–	–	Lineage II
SRR4897337	O157	stx1a/2c	–	–	Lineage II
SRR4897491	O157	–	–	–	Lineage II
SRR4897542	O157	stx2c	–	–	Lineage II
SRR5005528	O157	stx2c	–	–	Lineage II
SRR5006048	O157	stx2c	–	–	Lineage II
SRR5006078	O157	stx2c	–	–	Lineage II
SRR5006435	O157	stx2a/2c	–	–	Lineage II
SRR5017542	O157	–	–	–	Lineage II
SRR5024243	O157	–	–	–	Lineage II
SRR5024250	O157	stx2c	–	–	Lineage II
SRR5024470	O157	stx2c	–	–	Lineage II
SRR5024504	O157	stx2c	–	–	Lineage II
CP003109	O55	–	+	+	RM12579 SOR+ GUD+
SRR2034251	O157	stx2c	–	–	SOR–GUD– Stx2c
SRR4897272	O157	stx2c	–	–	SOR– GUD– Stx2c
SRR5033648	O55	–	–	+	SOR– GUD+ Stx2a Ireland
SRR5033651	O55	–	–	+	SOR– GUD+ Stx2a Ireland
SRR5033665	O55	–	–	+	SOR– GUD+ Stx2a Ireland
SRR5033691	O55	–	–	+	SOR– GUD+ Stx2a Ireland
SRR5033692	O55	–	–	+	SOR– GUD+ Stx2a Ireland
SRR5033714	O55	–	–	+	SOR– GUD+ Stx2a Ireland
SRR3241858	O55	stx2a	–	+	SOR– GUD+ Stx2a Outbreak
SRR5904128	O55	stx2a	–	+	SOR– GUD+ Stx2a Outbreak
SRR5023697	O55	stx2a	–	+	SOR– GUD+ Stx2a Outbreak
SRR3240983	O55	stx2a	–	+	SOR– GUD+ Stx2a Outbreak
SRR3240985	O55	stx2a	–	+	SOR– GUD+ Stx2a Outbreak
SRR3240986	O55	stx2a	–	+	SOR– GUD+ Stx2a Outbreak
SRR3241097	O55	stx2a	–	+	SOR– GUD+ Stx2a Outbreak
SRR3241840	O55	stx2a	–	+	SOR– GUD+ Stx2a Outbreak
SRR3241843	O55	stx2a	–	+	SOR– GUD+ Stx2a Outbreak
SRR3241872	O55	stx2a	–	+	SOR– GUD+ Stx2a Outbreak
SRR3242000	O55	stx2a	–	+	SOR– GUD+ Stx2a Outbreak
SRR3242001	O55	stx2a	–	+	SOR– GUD+ Stx2a Outbreak
SRR3578577	O55	stx2a	–	+	SOR– GUD+ Stx2a Outbreak
SRR3578601	O55	stx2a	–	+	SOR– GUD+ Stx2a Outbreak
SRR3581428	O55	stx2a	–	+	SOR– GUD+ Stx2a Outbreak
SRR5023710	O55	stx2a	–	+	SOR– GUD+ Stx2a Outbreak
SRR5023715	O55	stx2a	–	+	SOR– GUD+ Stx2a Outbreak
SRR5023716	O55	stx2a	–	+	SOR– GUD+ Stx2a Outbreak
SRR5023727	O55	stx2a	–	+	SOR– GUD+ Stx2a Outbreak
SRR5023769	O55	stx2a	–	+	SOR– GUD+ Stx2a Outbreak
SRR5023782	O55	stx2a	–	+	SOR– GUD+ Stx2a Outbreak
SRR5023783	O55	stx2a	–	+	SOR– GUD+ Stx2a Outbreak
SRR5023789	O55	stx2a	–	+	SOR– GUD+ Stx2a Outbreak
SRR5023803	O55	stx2a	–	+	SOR– GUD+ Stx2a Outbreak
SRR5023932	O55	stx2a	–	+	SOR– GUD+ Stx2a Outbreak
SRR5024312	O55	stx2a	–	+	SOR– GUD+ Stx2a Outbreak
SRR4897293	O157	stx2c	–	+	SOR– GUD+ Stx2c
SRR5017532	O157	stx2c	–	+	SOR– GUD+ Stx2c
SRR5018181	O157	stx2c	–	+	SOR– GUD+ Stx2c
AEUA01000000	O55	–	+	+	SOR+ GUD+
SRR4179257	O55	–	+	+	SOR+ GUD+ Ireland
SRR2187155	O157	stx2a	+	+	SOR+ GUD+ Stx2a
SRR5280511	O157	stx2a	+	+	SOR+ GUD+ Stx2a
SRR5904106	O55	stx2a	+	+	SOR+ GUD+ Stx2a Ireland

ACCESSION	SEROGROUP	STX	SOR	GUD	ANNOTATION
SRR5904127	O107	stx2a	+	+	SOR+ GUD+ Stx2a Ireland
SRR5033689	O55	–	+	+	SOR+ GUD+ Stx2a Ireland
SRR5033719	O55	–	+	+	SOR+ GUD+ Stx2a Ireland
SRR3578942	O55	stx2d	+	+	SOR+ GUD+ Stx2d
ERR197201	O55	–	+	+	TL–000132 SOR+ GUD+
ERR197199	O55	–	+	+	TL–000142 SOR+ GUD+
SRS702210	O55	–	+	+	USA 5905 SOR+ GUD+
ERR1138642	O55	–	+	+	ZH–1141 SOR+ GUD+

*GUD, β-glucuronidase; SOR, sorbitol; STEC, Shiga toxin–producing *Escherichia coli*; stx, Shiga toxin.
